# Arc capsid signaling between serotonergic and dopaminergic neurons sets sleep depth in *Drosophila*

**DOI:** 10.64898/2026.08.06.743357

**Published:** 2026-08-10

**Authors:** Andrew R. Butts, Kylie DeNiro, Sven Bervoets, Jason D. Shepherd, Sophie Jeanne Cécile Caron

## Abstract

Arc genes evolved from Ty3 retrotransposons and encode proteins that self-assemble into virus-like capsids that package and transfer RNA between cells. This capsid-forming property may mediate a form of intercellular signaling distinct from classical synaptic transmission, but whether it operates within a defined neuronal system to control an ongoing behavior remains unclear. Here we show that Arc-dependent capsid signaling links serotonergic and PAM neurons to set sleep depth in *Drosophila melanogaster* . Loss of *dArc* genes deepened and consolidated sleep, increasing both sleep depth and the arousal threshold to mechanical stimulation, while leaving the diurnal sleep pattern intact. *dArc1* was required in serotonergic neurons and, to a lesser extent, in PAM neurons, and both the loss-of-function phenotype and its rescue depended on capsid formation. Knocking down *dArc1* in Sas-expressing cells, or Ptp10D in PAM neurons, reproduced the deeper, more consolidated sleep of *dArc ^-/-^* flies, implicating the Sas-Ptp10D machinery in this signaling. These results identify Arc-dependent capsid signaling as a mechanism that links two modulatory neuronal populations to regulate an ongoing homeostatic behavior.

## Full Text Availability

The license terms selected by the author(s) for this preprint version do not permit archiving in PMC. The full text is available from the preprint server.

